# *De-novo* Upper Gastrointestinal Tract Cancer after Liver Transplantation: A Demographic Report

**Published:** 2020

**Authors:** E. M. Dobrindt, M. Biebl, S. Rademacher, C. Denecke, A. Andreou, J. Raakow, D. Kröll, R. Öllinger, J. Pratschke, S. S. Chopra

**Affiliations:** 1 *Department of Surgery, Charité – Universitätsmedizin Berlin, Berlin, Germany*; 2 *Department of Visceral, Transplant, Thoracic and Vascular Surgery, Universitätsklinikum Leipzig, Leipzig, Germany*

**Keywords:** Esophageal cancer, Immunosuppression, Squamous cell cancer, Esophagectomy

## Abstract

**Background:**

Immunosuppression is essential after liver transplantation (LT). It, however, increases the risk for cancer.

**Objective:**

To evaluate the prevalence and outcome of upper gastrointestinal (GI) tract cancer in LT patients and assess the perioperative risk of surgery for the upper GI malignancies post-LT.

**Methods:**

2855 patients underwent LT at our clinic from 1988 to 2018. 20 patients developed upper GI cancer. Data were retrospectively extracted from our database. Analysis included patients’ specific data, tumor histopathology and stage, the treatment given and survival.

**Results:**

23 patients developed upper GI malignancies (2 gastric and 18 esophageal cancers; 3 excluded), translating to a incidence of 26.4 per 100,000 population per year. All patients were male. 80% showed alcohol-induced cirrhosis before LT. Most of the tumors were diagnosed at a stage ≥III. 70% underwent surgery and 78.6% developed postoperative complications. One-year-survival was 50%. Total survival rate was 28.6% with a median follow-up of 10 months (range: 0–184).

**Conclusion:**

Upper GI malignancies are more common after LT compared to the general population. Men after LT, due to alcohol-induced liver cirrhosis, are at a higher risk. Upper GI surgery after LT can be safe, but the severe risk for complications and a poor survival require strict indications.

## INTRODUCTION

Orthotopic liver transplantation (LT) is the first-line therapy in patients with end-stage liver disease. The continuous improvement in monitoring and follow-up of these patients has constantly increased the survival rates after LT. Post-LT, almost all patients receive immunosuppressive therapy to avoid rejection and maintain a stable graft function. Immunosuppressive medications increase the risk of malignancies, as they may lead to direct damage of the host DNA that impairs the immune competence of the recipient [1, 2]. The general incidence of de-novo malignancies after LT ranges from 2.6% to 33.6% and is one of the leading causes of late mortality [2, 3]. Compared to the general population, the incidence of cancer after solid organ transplantation is 2.1–4.3 times higher [2,4]. Other common aspects like smoking, alcohol abuse, viral infections, age and rejection count as additional risk factors [2]. Skin cancer and hematological malignancies are the leading types of malignancies in transplant patients [2, 5, 6]. Previous studies analyzed the incidence of solid organ cancer and show higher standardized incidence ratios (SIR), especially for stomach (SIR range: 1.2–1.77) and esophageal cancer (SIR range: 1.92–6.7) [1, 2, 7-9]. 

This study was conducted to evaluate the prevalence and outcome of patients who developed upper gastrointestinal (GI) tract cancer post-LT. The perioperative risk of surgery of the upper GI tract post-LT was also analyzed.

## PATIENTS AND METHODS

Between January 1988 and July 2018, 2855 patients underwent LT at our center. Patients were monitored by routine check-ups at every 2–3 years. Data were extracted from the digital patient documentation system and from the archive data backup. We identified 23 patients with upper GI tract cancer; three were not treated for cancer diagnosis at out center and thus were excluded from analysis. Sex, age at diagnosis of cancer, age at LT, liver disease leading to LT, immunosuppression, comorbidities, body mass index (BMI) at the time of cancer diagnosis, histopathological tumor type, grading, staging, the treatment given and surgery, postoperative complications according Clavien-Dindo-Classification, 30- and 90-day mortality and the overall survival after the diagnosis of cancer were evaluated.

Subgroup analysis was performed for patients with and without alcoholic liver disease as indication for LT. Statistical analyses were performed with SPSS^®^ for Windows^®^ ver 25 (IBM, Armonk, NY, USA). Survival among patients with and without surgical treatment was compared. A p value <0.05 was considered statistically significant.

## RESULTS

We identified 23 patients with upper GI cancer after LT, translating into an incidence of 26.4 per 100,000 population per year. All patients were male; three were excluded. Demographic data are given in Table 1. The median age at the time of LT was 55.1 (range: 25–61) years. The median age at the time of cancer diagnosis was 61.0 (range: 50–73) years; 16 (80%) patients underwent LT due to alcohol-induced cirrhosis; 2 (10%) had hepatitis C; 1 (5.0%), a_1_-antitrypsin deficiency; and another an unknown liver disease. Two patients suffered from an HCC at the time of LT. Thirteen (65%) patients received calcineurin inhibitors for immunosuppression; 6 (30%), a combination of calcineurin inhibitors and mycophenolate; and one a calcineurin inhibitor plus mTor-inhibitor. Chronic kidney disease (50%), arterial hypertension (45%) and diabetes mellitus (45%) were the most frequent comorbidities; they are followed by coronary heart disease (25%). Eight (40%) patients had a positive history for nicotine abuse. The median BMI at the time of cancer diagnosis was 23.8 (range: 19–35) years.

**Table 1:** Demographic data. Frequencies are given in absolute numbers and percentage. Age is presented as median and (range). Body-mass-index is presented as mean (range) (n=20)

Variable	Statistics
Sex (male/female)	20 (100%)/—
Age at LT, yrs	55.0 (35–61)
Age at time of cancer diagnosis, yrs	61.0 (50–73)
Time since LT, yrs	7.5 (0–25)
Underlying disease for LT	
Alcohol-induced liver cirrhosis	16 (80%)
Hepatitis C	2 (10%)
a_1_-antitrypsine deficiency	1 (5%)
Unknown liver disease	1 (5%)
Hepatocellular carcinoma at time of LT	2 (10%)
Immunosuppression	
CNI	13 (65%)
CNI + MMF	6 (30%)
CNI + mTor	1 (5%)
Comorbidities	
Coronary heart disease/Myocardial infarct	5 (25%)
Arrhythmia	4 (20%)
Arterial hypertonia	9 (45%)
Diabetes mellitus	9 (45%)
Chronic kidney disease	10 (50%)
Nicotine abuse	8 (40%)
Body-mass index, kg/m^2^	23.8 (19–35)

Eighteen (90%) patients had esophageal cancer; 2 (10%) had gastric cancer (Table 2.). Thirteen (65%) patients had squamous cell cancer; five (25%), adenocarcinoma; and one, a neuroendocrine tumor. There was no cancer with grade G1; eight (40%) had grade G2; and seven (35%), grade G3. In five patients, the grading was missing. The lowest detected clinical tumor stage was UICC IIA; seven (35%) patients had stage UICC IV at the time of diagnosis. The tumor staging could not be ascertained in two patients. 

**Table 2:** Characteristics of the tumor (n=20)

Variable	n (%)
Clinical tumor type	
Gastric cancer	2 (10%)
Esophageal cancer	18 (90%)
Histopathological differentiation	
Squamous type cancer	13 (65%)
Adenocarcinoma	5 (25%)
Neuroendocrine tumor	1 (5%)
No data	1 (5%)
Grading	
G1	—
G2	8 (40%)
G3	7 (35%)
No data	5 (25%)
Stage	
UICC I	—
UICC II A	3 (15%)
UICC II B	2 (10%)
UICC III A	4 (20%)
UICC III B	2 (10%)
UICC IV	7 (35%)
No data	2 (10%)
Treatment given	
Palliative	6 (30%)
Surgical	14 (70%)

Fourteen (70%) patients underwent surgery at our clinic (Table 3). Six (30%) patients received conservative treatment (chemotherapy or radiochemotherapy). Two patients underwent preoperative radiation; one patient received preoperative combined radio-chemotherapy. We performed 10 Ivor-Lewis operations, one McKneown operation, and three gastrectomies. Three (21%) patients had no postoperative complications; three (21%) had life-threatening grade IV complications, according to the Clavien-Dindo-Classification. Most (36%) patients developed respiratory complications. One died during the postoperative course. The 30- and 90-day mortality rate was 7.1% and 14.3%, respectively, in the group after surgery. Six (43%) patients developed tumor recurrence with either metastases or peritoneal carcinosis after surgery. 

**Table 3:** Characteristics of surgical therapy (n=14)

Variable	n (%)
Type of surgery	
Total gastrectomy	3 (21%)
Ivor-Lewis esophagectomy	10 (71%)
McKeown esophagectomy	1 (7%)
Neoadjuvant chemotherapy	3 (21%)
Adjuvant chemotherapy	2 (14%)
Post-operative complications	
Surgical side infection	3 (21%)
Cardiovascular complication	2 (14%)
Respiratory complication	5 (36%)
Multiple organ disorder	3 (21%)
Clavien Dindo classification of post-operative complications	
I	1 (7%)
II	2 (14%)
III A	—
III B	2 (14%)
IV A	—
IV B	3 (21%)
V	1 (7%)
Tumor recurrence	6 (43%)
Peritoneal carcinosis	3 (21%)
Metastases	3 (21%)
30-day mortality	1 (7%)
90-day mortality	2 (14%)
5-year survival	2 (14%)

**Figure 1:**
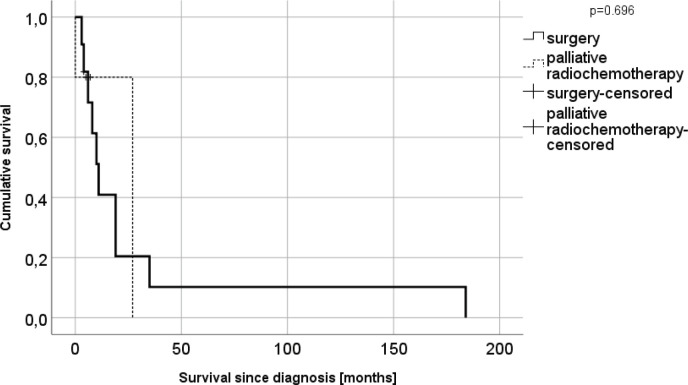
Survival since upper GI cancer diagnosis stratified by the treatment option (n=20)

**Figure 2:**
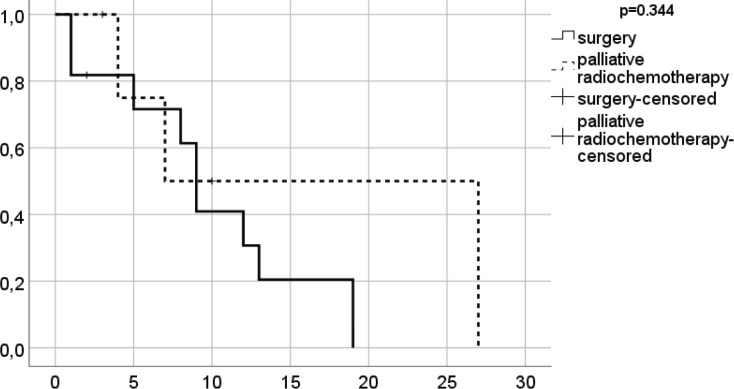
Survival since liver transplantation of patients with upper GI cancer stratified by the treatment option (n=20)

The mean follow-up from LT until cancer diagnosis was 8.7 (95% CI: 5.9–11.5) years in the total cohort. It was 12.0 (5.3–18.7) for adenocarcinomas; 7.6 (4.3–10.9), for squamous cell carcinomas; and 4.0, for neuroendocrine tumors (p=0.391, Fig 1). The post-LT survival, depending on the histopathological classification, was not significantly different with 13.5 (9.5–17.6) years in the total cohort; 15.2 (6.7–23.6), in patients with adenocarcinomas; 12.8 (8.9–16.7), for patients with squamous cell carcinomas; and 4 for the one patient with a neuroendocrine tumor (p=0.072, Fig 2).

The estimated mean overall survival was 26.2 (95% CI: 2.6–49.8) months since cancer diagnosis and 30.5 (0.0–65.1) in patients after surgery. Patients who received chemo- or radiochemotherapy, had an estimated mean overall survival of 21.6 (10.0–33.2) months; no significant difference was observed between the two groups (p=0.696, Fig 1). The estimated mean overall survival was 10.3 (76.2–14.5) years after LT in the total cohort; 9.6 (5.7–13.6), in patients after surgery; and 12.7 (0.0–26.8), in the palliative group (p=0.344, Fig 2). The survival rate was 29%. Demographic characteristics of patients with surgical or palliative therapy did not differ significantly for the parameters studied (Table 4).

**Table 4:** Clinical differences between surgically and palliatively treated patients. Values are either n (%) or mean (range).

Variable	Surgery (n=14)	Palliative (n=6)	p value
Age at LT, yrs	56.5 (39–61)	53.5 (35–60)	0.055
Age at time of cancer diagnosis, yrs	62 (54–73)	60 (56–66)	0.200
Time since LT, yrs	7.5 (0–23)	6.5(2–25)	0.200
Underlying disease for LT			0.039
Alcohol-induced liver cirrhosis	13 (93%)	3 (50%)	
Hepatitis C	—	2 (33%)	
a-1-antitrypsine deficiency	1 (7%)	—	
Unknown liver disease	—	1 (17%)	
Hepatocellular carcinoma at time of LT	2 (14%)	—	0.329
Immunosuppression			0.061
CNI	11 (79%)	2 (33%)	
CNI + MMF	2 (14%)	4 (67%)	
CNI + mTor	1 (7%)	—	
Nicotine abuse	6 (43%)	2 (33%)	0.690
Body mass index, kg/m^2^	23.8 (21–35)	23.1 (19–26)	0.018
Histopathological differentiation			0.158
Squamous type cancer	10 (71%)	3 (50%)	
Adenocarcinoma	4 (29%)	1 (17%)	
Neuroendocrine tumor	—	1 (17%)	
No data	—	1 (17%)	
Tumor stage			0.405
UICC I	—	—	
UICC II A	2 (14%)	—	
UICC II B	2 (14%)	—	
UICC III A	3 (21%)	1 (17%)	
UICC III B	2 (14%)	1 (17%)	
UICC IV	3 (21%)	4 (67%)	
No data	2 (14%)		

**Figure 3:**
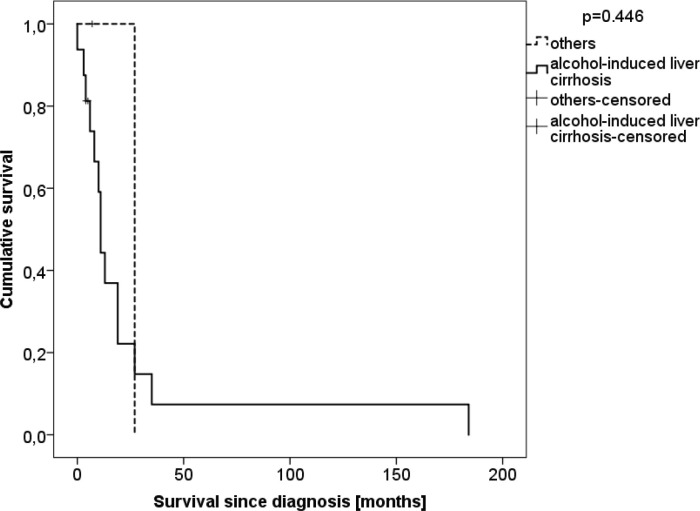
Survival since upper GI cancer diagnosis of patients stratified by alcohol abuse as the cause of transplantation (n=20)

**Figure 4:**
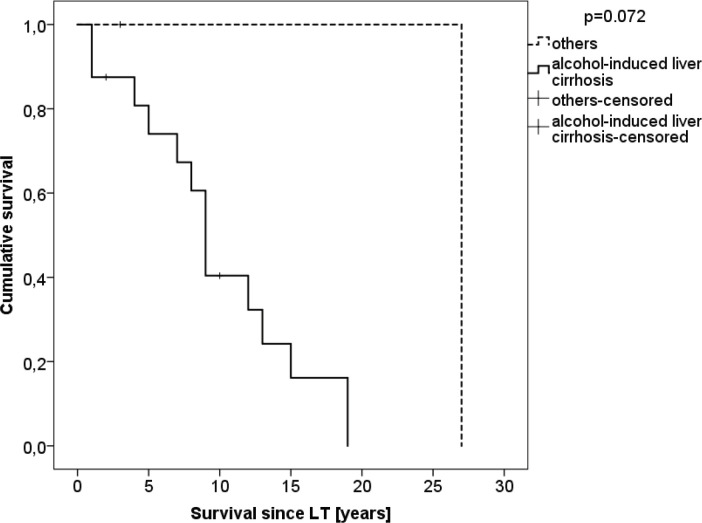
Survival since liver transplantation of patients with upper GI cancer stratified by alcohol abuse as the cause of transplantation (n=20)

Subgroup analysis was performed for patients with and without “alcoholic liver disease as their underlying disease for LT” (Table 5). There was no significant difference between both groups in terms of age at LT or diagnosis, time of follow-up, HCC at the time of LT, immunosuppression, BMI, clinical or histopathological type of cancer, as well as the treatment given and survival. The estimated mean overall survival since cancer diagnosis was 25.8 (95% CI: 0.7–50.9) months in patients with alcoholic liver disease and 27.0 (0.0-27.0) in the control group (p=0.446, Fig 3). The estimated mean overall survival since LT was 9.9 (6.9–12.8) months in patients with alcoholic liver disease and 27.0 (27.0–27.0) in the control group (p=0.072, Fig 4). 

**Table 5:** Subgroup analysis for patients with and without alcoholic liver disease as the underlying indication for transplantation. Values are either n (%) or mean (range).

Variable	Alcohol-induced liver cirrhosis (n=16)	Others (n=4)	p value
Age at LT, yrs	55.5 (43–61)	48.0 (35–60)	0.437
Age at the time of cancer diagnosis, yrs	61.5 (56–73)	62.0 (54–66)	0.892
Time since LT, yrs	7.0 (0–23)	11.5(2–25)	0.335
Hepatocellular carcinoma at time of LT	2 (12.5%)	—	0.632
Immunosuppression			0.088
CNI	12 (75%)	1 (25%)	
CNI + MMF	3 (19%)	3 (75%)	
CNI + mTor	1 (6%)	—	
Nicotine abuse	7 (44%)	1 (25%)	0.619
Body mass index, kg/m^2^	23.6 (21–35)	22.1 (19–26)	0.549
Clinical tumor type			1.000
Gastric cancer	2 (13%)	—	
Esophageal cancer	14 (88%)	4 (100%)	
Histopathological differentiation			0.081
Squamous type cancer	12 (75%)	2 (50%)	
Adenocarcinoma	3 (19%)	1 (25%)	
Neuroendocrine tumor	1 (6%)	—	
No data	—	1 (25%)	
Tumor stage			0.582
UICC I	—	—	
UICC II A	2 (13%)	1 (25%)	
UICC II B	2 (13%)	—	
UICC III A	3 (25%)	—	
UICC III B	2 (13%)	—	
UICC IV	5 (31%)	2 (50%)	
No data	1 (6%)	1 (25%)	
Treatment for upper GI cancer			0.061
Surgery	13 (81%)	1 (25%)	
Palliative	3 (19%)	3 (75%)	

## DISCUSSION

Upper GI cancer has an incidence of 26.4 per 100,000 population per year in our transplant cohort (n=2855). This is in agreement with the range reported in general population, i.e., 4–30 per 100,000 population per year during the first decade of the 20^th^ century [5, 10, 11]. The proportion of squamous cell carcinoma was higher than the adenocarcinoma, according to the general population [10]. Several common risk factors are described for esophageal cancer. Those include sex, older age, race, alcohol and nicotine abuse, adipositas, and reflux disease [10, 12-14]. All patients with cancer in our study were male. Because there was a higher percentage of men in our transplant cohort (60.1%), that finding might be biased. Nowever, males carry a significantly higher risk [12,14]. Alcohol is another independent risk factor for esophageal cancer [10, 11]. Most of the patients included in the analysis presented with alcohol-induced liver cirrhosis as their underlying disease for LT (95.6%). Patients had to fulfill abstinence at the time of LT, however, relapse is estimated at up to 16% [15]. As alcohol abuse, especially after LT, remains a stigma, capturing adequate data persists challenging and there might be a relevant number of unreported cases [14]. We could not deliver numbers of active drinkers after LT. Nonetheless, previous studies show that patients with alcohol-induced liver disease and alcohol abuse prior to LT have a significant higher risk for malignancies, especially esophageal cancer, post-LT [1, 14, 16]; 80% of the diagnosed patients suffered from alcohol-induced liver failure; 40% of patients presented with additional nicotine consumption, another well-known risk factor for upper GI cancer [12, 17]. 

A Danish study revealed that male patients with abusive alcohol consumption carry a 4.1-fold increase in the risk of developing esophageal cancer compared with the general population; 1.4-fold increase, for stomach cancer [18]. The National German Cancer Registry estimated an incidence of 14.2 cases per 100,000 male population in 2018 [19]. Regarding the alcohol-associated risk for esophageal cancer compared with the standardized risk of the general population, our patients still carry a nearly 14-fold increase in risk of developing malignancies of the upper GI tract. According to our subgroup analysis, we performed a comparison of patients with and without alcoholic liver disease as the underlying cause of LT. We found significant difference in terms of demographic or survival data. However, considering a very small control group, this finding should be interpreted with caution. Beside the general risk factors, there is a doubled risk for de novo malignancies after LT [16]. Immunosuppression is thought to impair cancer surveillance mechanisms and support the environment for oncogenic viruses [16]. We showed that the incidence rate for upper GI tract malignancies is higher than expected after LT, even after consideration of additional risk factors. Therefore, a strict follow-up and clinical monitoring are necessary after LT as general prevention and screening procedures might be insufficient to ensure at least an early diagnosis. Rousseau,* et al*, could show that adequate optimal oncologic treatment is feasible in 180 recipients after kidney or liver transplantation without any safety concerns even if prognosis and oncologic treatment of de novo cancer in solid organ transplant patients remain poorly described [1, 20]. Modification of immunosuppressive therapy might be necessary and especially the introduction of mTor-inhibitor can improve survival [18]. 

Besides interdisciplinary strategies, most of the patients will need surgical procedures. Intra-abdominal adhesions after LT may impede surgical procedures and extend the operation time. In addition, immunosuppression impairs healing and increases the risk for surgical-associated infections and anastomotic insufficiency [21-23]. Therefore, the question of the individual surgical risk for major surgery like esophagectomy or gastrectomy has to be answered in every individual case. 

There are heterogenous reports about the influence of immunosuppression in patients with Morbus Crohn undergoing bowl surgery [24-32]. Most studies analyzed the influence of corticoids and antibodies, but there is a lack of information about calcineurin inhibitors. Calcineurin inhibitors were the most common immunosuppressive agent in our cohort; 78.6% of the patients developed post-operative complications; 21.4% of them had life-threatening complications of grade IV, according to Clavien-Dindo classification. Current literature reports about lower complication rates of 25%–60% after esophagectomy in the general population [33, 34]. Pneumonia and atrial dysrhythmia seem to be the most common complications, followed by anastomotic leakage [33]. In our cohort, we could confirm respiratory complications (35.7%) as the most frequent complication with a higher incidence compared to former studies (14.6%–25%) [33, 34]. This might be a side effect of immunosuppression. In contrary, we did not detect an anastomotic leak in our cohort, which was lower compared with standard patients treated in larger centers (10%–13%) [33]. The 30- and 90-day mortality rates were 7.1% and 14.3%, respectively; the overall mortality was 71.4% during a median follow-up of up to 10 months (range: 0–184). Current studies showed a 30-day mortality between 0.73% and 3.8% for esophagectomy. In-house mortalities were 5.49%–8.3%. Only 20%–30% survived the first 5 years [10, 33, 35-39]. The 5-year survival rate was 14.3% in the surgical group of our cohort; it was lesser than previous reports. The palliative group included only six patients with synchronous metastases. The survival of these patients was generally lower. 

Survival is also influenced by comorbidities. Most of the patients had severe comorbidities. One patient, who died during the postoperative course, presented with severe cardiac and pulmonary comorbidity. He had received radiation due to lung cancer prior to the diagnosis of esophageal cancer; surgical treatment was thus the only option. This patient consciously asked for surgical treatment.

Regarding the high mortality and morbidity after esophagectomy and gastrectomy due to upper GI cancer, the indication strictly depends on the patient’s comorbidities and constitution. However, we found that major surgery of the upper GI tract cancers is safe even after LT and might improve patient’s survival in comparison with non-surgical treatments. Modern techniques like minimal invasive surgery or robotic-assisted surgery can help to minimize post-operative complications and allow fast tract concepts even in critical patients [40]. The main burden after operation is respiratory complications and should be pre-emptively treated by pre-habilitation, antibiotic therapy, and intensive breathing therapy. As shown, patients after LT suffer from a higher incidence of de novo malignancies and upper GI cancer. Those tumors may need surgical treatment. Major surgical resections should not be denied to those patients fearing the complications after prior LT and can be offered after conscious risk evaluation. Screening protocols might be adapted for this patient group to allow earlier diagnosis and provide a better prognosis.

The limitation of this study was the retrospective nature of the study and small number of patients studied. Upper GI tract cancer remains a rare entity even in transplant patients and large, prospective and blinded studies are difficult to conduct. Nonetheless, further investigations are necessary to evaluate the influence of immunosuppression on the outcome of major surgery and post-operative complications. This analysis should provide aspects from the clinical praxis to show that surgery for upper GI cancer after LT can be performed.
